# The Role of AP2/ERF Transcription Factors in Plant Responses to Biotic Stress

**DOI:** 10.3390/ijms26104921

**Published:** 2025-05-21

**Authors:** Ze-Lin Su, Ao-Mei Li, Miao Wang, Cui-Xian Qin, You-Qiang Pan, Fen Liao, Zhong-Liang Chen, Bao-Qing Zhang, Wen-Guo Cai, Dong-Liang Huang

**Affiliations:** 1 College of Life Science and Technology, Guangxi University, Nanning 530004, China; 2 Guangxi Key Laboratory of Sugarcane Genetic Improvement, Key Laboratory of Sugarcane Biotechnology and Genetic Improvement (Guangxi), Sugarcane Research Institute, Guangxi Academy of Agricultural Sciences, Nanning 530007, China

**Keywords:** AP2/ERF, transcription factors, molecular breeding, biotic stress, regulation expression

## Abstract

The APETALA2/ethylene response factor (AP2/ERF) family of transcription factors (TFs) is one of the largest and most important TF families in plants. This family plays a crucial role in regulating growth, development, and responses to both biotic and abiotic stresses. This study provides a comprehensive overview of the structure, classification, and distribution of AP2/ERF TFs in various plant species, with particular emphasis on their roles in responses to biotic stress. These findings provide valuable insights for future research on AP2/ERF TFs and their potential applications in crop improvement through molecular breeding.

## 1. Introduction

Transcription factors (TFs) are defined as DNA-binding proteins that specifically bind to cis-acting elements within promoter regions. The expression of target genes is regulated by TFs at specific times and in specific locations with a certain intensity, resulting in the activation or repression of gene transcription [1]. To date, approximately 60 TF families have been identified in plants, including AP2/ERF, bZIP, C2H2, MYB, MADS, NAC, and WRKY [2].

The APETALA2/ethylene response factor (AP2/ERF) family of TFs is one of the largest and most significant important TF families in plants and is characterized by at least one AP2 domain of approximately 60 amino acids. This family is extensively involved in plant growth and development and in responses to biotic and abiotic stresses [3,4,5]. The AP2/ERF TFs include five subfamilies. The APETALA2 (AP2) subfamily plays crucial roles in regulating various aspects of plant growth and development, such as flower development [6,7,8], leaf morphology [9], epidermal-cell identity in leaves [10], spikelet-meristem determinacy [11], embryonic development [12], and seed growth [13]. The ethylene response factor (ERF) subfamily is essential for hormone signal transduction [14,15], responses to biotic and abiotic stresses [16,17], plant development [18], and metabolic regulation [19,20,21]. The dehydration-responsive-element-binding (DREB) subfamily is primarily associated with supporting the plant’s responses to abiotic stresses, specifically drought and cold stresses [22,23]. The related to ABI3/VP1 (RAV) subfamily plays important roles in regulating leaf senescence [24], ethylene (ET) [25] and brassinosteroid (BR) signaling [26], and responses to biotic and abiotic stresses [27]. However, the functional characterization of the soloist subfamily remains limited, and its regulatory mechanisms and biological significance are not well understood. Although numerous reviews on AP2/ERF have been published, few studies have focused on its role in plant responses to biotic stress. Recent research findings together have clarified the regulatory network of the AP2/ERF family in response to biotic stress and have shown that it involves hormone signaling pathways, MAPK cascades, cell-wall reinforcement, and epigenetic regulation. These efforts provide a theoretical basis and technical support for the development of stress-resistant and high-yield crop varieties, thereby contributing to the advancement of sustainable agriculture.

## 2. The Structure and Classification of AP2/ERF TFs

**Figure 1:**
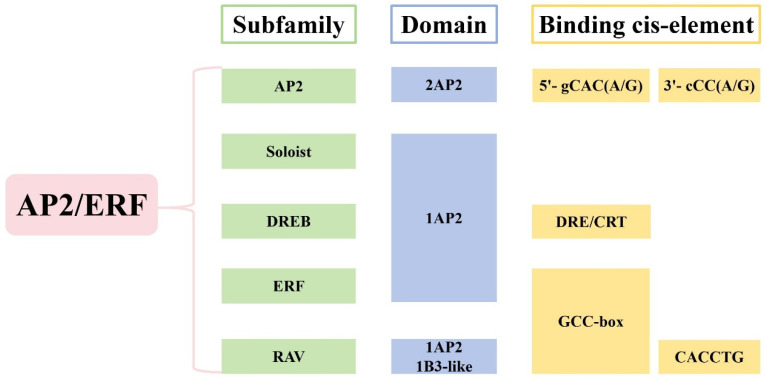
The structure and classification of AP2/ERF TFs.

The AP2/ERF TFs are characterized by the AP2 domain, a DNA-binding motif of approximately 60–70 amino acid residues [28]. Sakuma et al. classified the AP2/ERF TFs into five subfamilies (AP2, ERF, DREB, RAV, and Soloist) based on the number of AP2 domains and other structural features [3]. All AP2/ERF-domain sequences contain a single α-helix and three β-sheets [29]. The earliest identified subfamily, AP2, contains two AP2 domains [30]. The ERF subfamily was first isolated from tobacco by Ohme-Takagi et al. in 1995 and contains a single AP2 domain [31]. The DREB subfamily was initially identified in *Arabidopsis thaliana* by Stockinger et al. [32]. Like the ERF subfamily, the DREB subfamily contains only a single AP2 domain. However, the AP2-domain amino acid residues at positions 14 and 19 differ between DREB and ERF TFs. The RAV subfamily, first reported by Kagaya et al. in 1999, contains both an AP2 domain and a B3-like domain [33]. The Soloist subfamily also contains an AP2 domain, but its amino acid motif and conformation are significantly distinct from those of other subfamily members. Nakano et al. proposed an alternative classification by merging the DREB and ERF subfamilies into a single group, categorizing AP2/ERF TFs into four classes [34]. Both classification methods are widely used in the literature as primary approaches to AP2/ERF TF family classification (Figure 1).

Due to differences in the amino acid sequences and nuclear-localization signals of its two AP2 domains, the AP2 subfamily can be further divided into the AP2 and ANT groups [35]. Nole-Wilson et al. proposed that the first AP2 domain of ANT binds to the 5′-gCAC(A/G) sequence, whereas the second domain binds to the 3′-cCC(A/G) sequence [36]. Members of the AP2 subfamily are primarily involved in regulating processes such as flowering and lateral root formation in plants. Previous studies collectively referred to the ERF and DREB subfamilies as the ERF subfamily. Later, based on the cis-acting elements bound by ERF transcription factors, researchers divided this group into the ERF and DREB subfamilies [37]. Members of the ERF subfamily bind the ethylene-responsive GCC-box element and regulate ethylene signaling and responses to abiotic stresses [38]. The DREB subfamily binds to drought- and cold-stress-responsive elements (DRE/CRT), thereby inducing expression of related genes and enhancing plant tolerance to abiotic stresses [22,23]. The key distinction between the ERF and DREB subfamilies lies in the amino acid residues at the 14th and 19th positions within their AP2/ERF domains. Within the ERF subfamily, the 14th and 19th residues are alanine and aspartic acid, respectively, whereas in the DREB subfamily, they are valine and glutamic acid [37]. This difference in residues determines each transcription factor’s specific binding to distinct cis-acting elements. In the RAV subfamily, an AP2/ERF domain at the N-terminus binds the GCC-box element, whereas the B3 domain at the C-terminus specifically binds the CACCTG sequence [39,40].

## 3. The Distribution and Quantity of AP2/ERF TFs Involved in Responses to Biotic Stresses 

Advances in technologies such as whole-genome sequencing have led to the identification of an increasing number of AP2/ERF TFs in plants. Within the AP2/ERF TF family, the ERF and DREB subfamilies have the most members, followed by the AP2 subfamily, while the RAV and Soloist subfamilies contain the fewest [41,42,43]. The AP2/ERF TF family has now been identified in various plant species. For example, *Arabidopsis thaliana* has 122 ERF genes, 18 AP2 genes, 6 RAV genes, and 1 Soloist gene [34]. In sorghum, 105 ERF genes, 16 AP2 genes, 4 RAV genes, and 1 Soloist gene have been reported [44]. Similarly, buckwheat contains 116 ERF genes, 15 AP2 genes, and 3 RAV genes [45].

**Figure 2:**
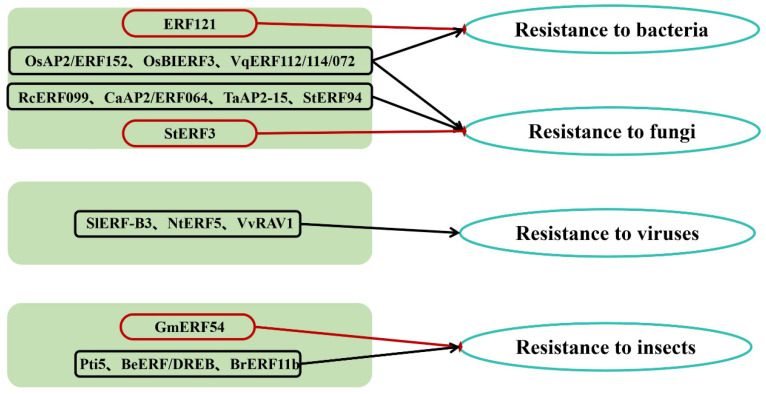
The role of AP2/ERF in biotic stress.

AP2/ERF TFs typically activate the expression of downstream genes responsive to biotic stress, thereby enhancing plant resistance to various pathogens and insect pests (Figure 2 and Table 1) [46,47]. Biotic stresses, such as bacterial, fungal, and viral infections, as well as insect pests, can lead to crop-yield reduction or even plant death [48,49].

### 3.1. Bacterial Diseases

Bacterial diseases affect a wide range of plants, causing various symptoms such as leaf spots, blights, wilts, and cankers. Pillai et al. demonstrated that overexpression of the *OsAP2/ERF152* gene in *Arabidopsis thaliana* induced callose deposition and other immune responses, thereby enhancing resistance to bacterial and fungal infections [50]. Overexpression of the rice *OsBIERF3* gene enhances resistance to both fungal and bacterial pathogens [52]. Additionally, *VqERF112*, *VqERF114* and *VqERF072* from Chinese wild grape (*Vitis quinquangularis*) positively regulate resistance to *Pseudomonas syringae pv. tomato* DC3000 and *Botrytis cinerea* [53]. Conversely, in *Brassica oleracea*, the *ERF121* TF increases susceptibility to *Xanthomonas* infections by disrupting plant defense pathways [51].

### 3.2. Fungal Diseases

Fungal diseases, caused by pathogenic fungi, are the most prevalent and destructive plant diseases, comprising over 80% of all known plant diseases. There are two main types of fungal pathogens: biotrophs and nectrotrophs. Together with bacteria, they cause vascular wilts, leaf spots, cankers, and other symptoms, infecting various parts of the plant. Biotrophic pathogens first penetrate epidermal cells and multiply in the intercellular spaces by feeding on living host tissue. Most biotrophs, including *Pseudomonas syringae*, are host-specific. Necrotrophic pathogens kill host-plant cells using toxic metabolites and then feed on the remains. Most necrotrophs infect a wide range of hosts. *RcERF099* is an important positive regulator of resistance to *Botrytis cinerea* infections in rose petals [54]. Overexpression of *CaAP2/ERF064* enhances resistance to Phytophthora blight in *Capsicums* (peppers) [55]. Silencing *TaAP2-15* increases susceptibility to *Puccinia striiformis f. sp. tritici* in wheat, promoting pathogen growth [56]. In potato, overexpression of *StERF94* inhibits fungal proliferation in cellular tissues, thereby enhancing disease resistance [57]. Conversely, *StERF3* negatively regulates resistance to *Phytophthora* infestans, the causal agent of late blight [58].

### 3.3. Viral Diseases

Viral diseases cause not only local lesions but also systemic damage that leads to stunting, chlorosis and malformations affecting different parts of the plant, although they rarely kill their hosts. *Tomato yellow leaf curly virus* (TYLCV) causes leaf curling and yellowing, plant dwarfism, and growth inhibition in tomato. A comprehensive analysis revealed that five *SlERF-B3* TFs are differentially expressed in resistant and susceptible tomato during TYLCV infection [59]. In tobacco, overexpression of *NtERF5* enhances resistance to *tobacco mosaic virus* [60]. Grapevine leafroll-associated virus 2 (GLRaV-2) is a common virus associated with grapevine leafroll disease. The *GLRaV-2* p24 protein interacts with *VvRAV1*, thereby suppressing host defense responses and facilitating accumulation and infection of *GLRaV-2* in grape [61].

### 3.4. Insect Pests

Insect pests often damage plants through feeding or by transmitting plant pathogens, reducing growth and yield. Ulti-host common cutworm (CCW) is a major herbivore in low latitudes of China which feeds almost all soybean tissues [78]. Overexpression of *GmERF54* decreases resistance to CCW in transgenic soybean [62]. In herbivore-induced defense responses, expression of *Pti5* is induced by potato aphid feeding, and the ERF *Pti5* gene contributes to resistance to potato aphids in tomato [63]. Bambusa emeiensis is one of the most economically important bamboos, and its BeERF/DREB subfamily plays a critical role inresponding to herbivore attack [79]. *BrERF11b* enhances plant resistance to both chewing and sap-sucking insects [65].

## 4. Regulation of Hormones in Responses to Biotic Stress

**Figure 3:**
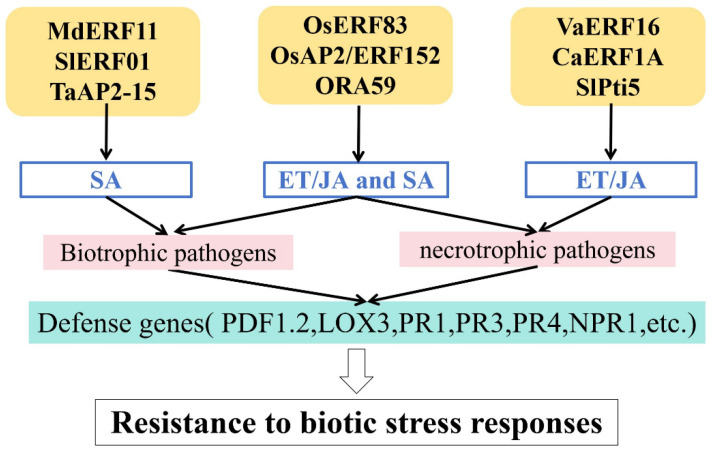
Regulation of hormones in responses to biotic stress.

The AP2/ERF TF family serves as a pivotal regulatory hub connecting plant hormone signaling pathways (Figure 3 and Table 1). They induce plant hormone responses by activating target genes or acting as response factors. Current research indicates that AP2/ERF TFs primarily regulate responses to biotic stress via plant hormone signaling pathways such as those mediated by ethylene (ET), jasmonic acid (JA), and salicylic acid (SA) [80,81].

### 4.1. SA-Mediated Regulation

SA is a plant hormone involved in defense signaling; it activates pathogenesis-related (PR) genes, thereby modulating responses to biotic stress. The SA signaling pathway confers resistance to biotrophic pathogens [82,83].

Wang et al. showed that *MdERF11* enhances resistance to gray mold disease in apple trees by promoting the expression of genes involved in SA synthesis [66]. Yang et al. reported that expression of *SlERF01* is strongly induced by *Stemphylium lycopersici* and exogenous applications of SA, thereby enhancing resistance to *Stemphylium lycopersici* in tomato [67]. *TaAP2-15* acts as a positive regulator of resistance to *Puccinia striiformis f.* sp. *tritici (Pst)* in wheat via an SA-induced mechanism [56].

### 4.2. ET/JA-Mediated Regulation

In addition to SA, AP2/ERF TFs regulate disease resistance through the ET/JA signaling pathway. The ET/JA signaling pathways are particularly effective against necrotrophic pathogens such as *Botrytis cinerea* and *Fusarium oxysporum* [84,85]. *VaERF16* acts as a positive regulatory factor by enhancing the ET/JA-signaling-dependent response to hormone signals, thereby improving resistance to *Botrytis cinerea* in grape [68]. In hot pepper, overexpression of *CaERF1A* enhances resistance to necrotrophic fungal pathogens by upregulating an ET/JA-synthesis-related gene [69]. Silencing of the *SlPti5* gene in tomato weakens the ET/JA signaling pathway, impairing immune responses and reducing resistance to *Botrytis cinerea* [64].

### 4.3. ET/JA- and SA-Mediated Regulation

More importantly, AP2/ERF TFs mediate crosstalk between the SA and JA/ET signaling pathways. Tezuka et al. demonstrated that treatment with ET, JA, and SA induces *OsERF83* expression, thereby enhancing the expression of PR genes in transgenic rice and significantly improving resistance to rice blast disease [70]. Pillai et al. found that expression of *OsAP2/ERF152* upregulates SA- and JA/ET-responsive defense genes, enhancing resistance to bacterial and fungal pathogens in *Arabidopsis thaliana* [50]. In *Arabidopsis thaliana*, *ORA59* is a key positive regulator of the ET/JA-mediated defense pathway that protects against necrotrophic pathogens, but it is repressed by SA signaling [71].

## 5. Regulation of MAPK in Responses to Biotic Stress

In plants, MAPKs generally act downstream of sensors/receptors that recognize endogenous stimuli or exogenous stimuli, coordinating plant growth, development, and immunity. Activated MAPKs mediate the phosphorylation of various downstream substrates, such as protein kinases, TFs, structural proteins, and other enzymes, to activate cellular responses [86,87].

**Figure 4:**
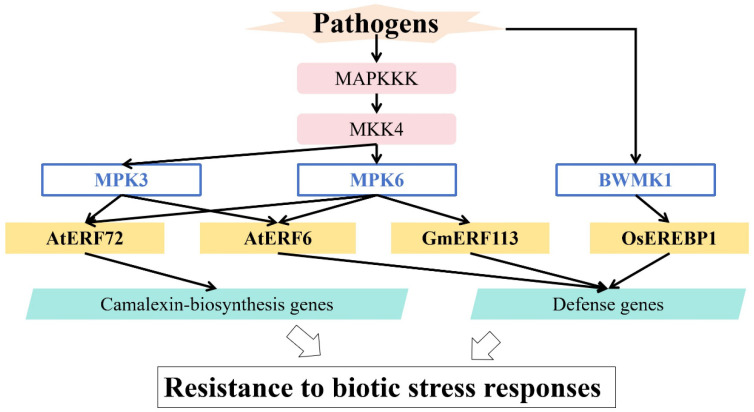
Regulation of MAPK in responses to biotic stress.

Multiple AP2/ERF TFs have been shown to be substrates of MAPKs, linking them to plant defense responses. MAPK cascades are important signaling modules in responses to biotic stress (Figure 4 and Table 1). In *Arabidopsis thaliana*, the partially redundant MAPKs MPK3/MPK6 phosphorylate At*ERF6* and At*ERF72*, enhancing resistance to *Botrytis cinerea* via different pathways [72,73]. In rice, MAP kinase BWMK1 phosphorylates *OsEREBP1*, promoting its binding to the GCC-box in PR gene promoters and thus enhancing PR gene expression and disease resistance [74]. Moreover, the soybean GmMKK4-GmMPK6 module phosphorylates *GmERF113*, promoting the stability of its protein product and enhancing transcriptional activity; it thereby increases the expression of defensive genes and enhances immune responses to *Phytophthora sojae* [75].

## 6. Regulation of Cell Wall in Responses to Biotic Stress

In plants, the cell wall is a physical barrier against adverse stress and pathogen invasion, and it plays a crucial role in signaling under conditions of biotic stress [88,89]. Lignin, a key component of the cell wall, enhances the wall’s defensive capabilities [90,91].

**Figure 5:**
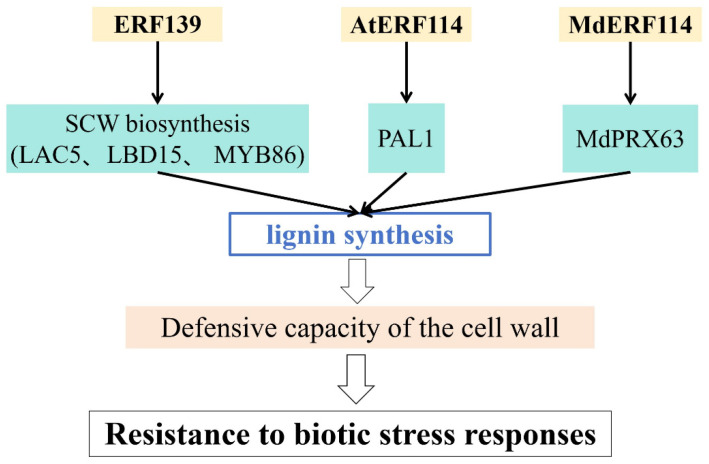
Regulation of the cell wall in responses to biotic stress.

Multiple investigations have reported that AP2/ERF TFs critically regulate plant defenses by altering lignin biosynthesis (Figure 5 and Table 1). For example, *ERF139* in *Populus* plays a role in the lignin-synthesis pathway by inducing the expression of genes related to secondary cell-wall synthesis, thereby enhancing resistance to biotic stress [76]. In *Arabidopsis thaliana*, *AtERF114* activates the lignin biosynthetic gene *AtPAL1* by directly binding to its promoter, increasing lignin accumulation and enhancing immunity [77]. Similarly, *MdERF114* promotes lignin accumulation by regulating the expression or transcriptional activity of *MdPRX63*, resulting in enhanced lignin deposition and disease resistance [48].

## 7. Epigenetic Regulation of the AP2/ERF TF Family in Plant Growth and Development

**Figure 6:**
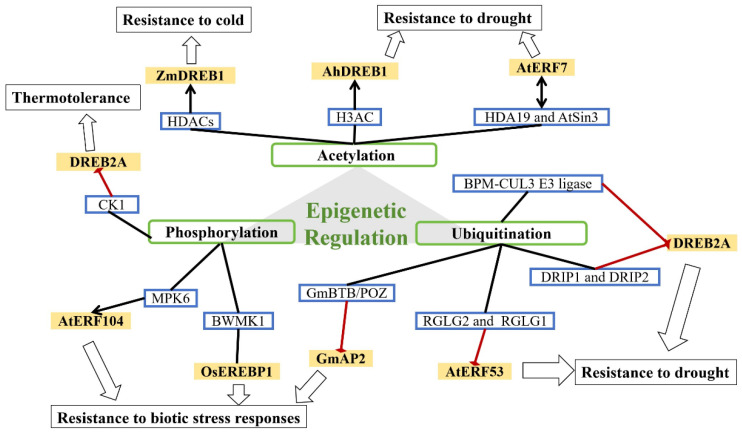
Epigenetic regulation of the AP2/ERF TF family in plant growth and development.

Epigenetic regulation plays a significant role in plant growth and development [92]. It primarily affects DNA methylation and histone modifications (including methylation, acetylation, phosphorylation, and ubiquitination) [93,94]. Current research on AP2/ERF TFs has mainly focused on histone modifications (Figure 6).

The level of histone acetylation is controlled by the dynamic balance between histone acetyltransferases (HATs) and histone deacetylases (HDACs) [95]. In maize, HDACs cause global histone deacetylation, promoting the expression of *ZmDREB1* under cold stress [96]. In peanut, histone acetylation regulates *AhDREB1* under conditions of osmotic stress by increasing H3ac levels, thereby improving drought resistance [97]. Song et al. showed that in *Arabidopsis thaliana*, *AtERF7* interacts with the transcriptional corepressor AtSin3, which is associated with histone deacetylase 19 (HDA19). HDA19 and AtSin3 enhance the transcriptional-repression activity of *AtERF7*. Overexpression of *AtERF7* in transgenic *Arabidopsis thalian* decreases drought tolerance [98].

Phosphorylation modulates AP2/ERF protein transactivity and stability. Phosphorylation by different kinases usually results in a functional change in the target protein by changing activity, cellular location, or association with other proteins. For example, the rice MAP kinase BWMK1 phosphorylates *OsEREBP1*, enhancing its DNA-binding activity to the GCC box element and activating expression of pathogenesis-related genes [74]. Phosphorylation by MPK6 releases *AtERF104*, which in turn activates defense genes such as PDF1.1 and PDF1.2 [99]. Under nonstress conditions, the negative-regulatory domain (NRD) of *DREB2A* is phosphorylated primarily by casein kinase 1 (CK1), which facilitates *DREB2A* degradation and thereby negatively regulates its stability [100].

Ubiquitination may affect proteins in various ways, including targeting them for proteasome-mediated degradation, altering cellular localization, modifying their activity, and enhancing or inhibiting protein–protein interactions. In *Arabidopsis*, BTB/POZ–MATH (BPM) adaptor proteins of the CUL3 E3 ligase complex mediate *DREB2A* degradation to coordinate drought-stress responses [101,102]. Similarly, DRIP1 and DRIP2 function as E3 ubiquitin ligases that ubiquitinate *DREB2A*, targeting it for degradation via the 26S proteasome and thus negatively regulating the drought response [103]. RGLG2 and its closest homolog, RGLG1, have E3-ligase activity, mediating *AtERF53* ubiquitination and degradation via the 26S proteasome pathway and thus negatively regulating drought-stress responses [104]. In soybean, the GmBTB/POZ protein promotes ubiquitination and degradation of the AP2/ERF-like TF *GmAP2*, thereby regulating the defense response against *Phytophthora sojae* [105].

## 8. Challenges and Future Directions for AP2/ERF TFs

AP2/ERF TFs are ubiquitous in plants and play critical roles in mediating resistance to diverse biotic stresses, including bacterial, fungal, viral, and insect challenges. Since the cloning of the first AP2/ERF TFs in *Arabidopsis thaliana*, substantial progress has been achieved in elucidating their functions; however, many questions remain to be resolved. To date, research on the roles of AP2/ERF TFs in responses to biotic stress has largely focused on plants such as *Oryza sativa* and *Arabidopsis thaliana*, whereas studies in other plants have concentrated on responses to abiotic stresses. AP2/ERF TFs are predominantly characterized as positive regulators, whereas their negative regulatory functions remain underexplored. Therefore, novel AP2/ERF family members that regulate multiple facets of plant responses to biotic stress, as well as their underlying mechanisms, warrant exploration in future research.

Additionally, with the advancement of molecular biology and high-throughput sequencing technologies, genetic engineering, such as the use of CRISPR/Cas9 to breed resistant varieties, has arisen as a new method for improving some crops. Therefore, future research can build on existing methodologies by using molecular-biology techniques to investigate AP2/ERF TFs and their roles in responses to biotic stress in a wider range of plants. Key areas of focus should include the similarities and differences in the regulatory mechanisms of AP2/ERF TFs in various responses to biotic stress, their roles in coordinating multiple signaling pathways, and the mechanisms though which they exert their effects. With the identification of novel AP2/ERF TFs and the elucidation of the underlying mechanisms, AP2/ERFs may serve as promising targets in breeding for disease resistance in many crops.

## Figures and Tables

**Table 1:** AP2/ERF TFs involved in different responses to biotic stresses.

Species	Gene Name	Subfamily	Function	Reference
*Oryza sativa*	*OsAP2/ERF152*	AP2/ERF	Enhances resistance to bacterial and fungal infections	[50]
*Brassica oleracea*	*ERF121*	ERF	Promotes susceptibility to *Xanthomonas* infections	[51]
*Oryza sativa*	*OsBIERF3*	ERF	Enhances resistance to fungal and bacterial pathogens	[52]
*Vitis quinquangularis*	*VqERF112/114/072*	ERF	Positively regulates resistance to *Pseudomonas syringae pv. Tomato* DC3000 and *Botrytis cinerea*	[53]
*Rose chinensis*	*RcERF099*	ERF	Positively regulates resistance to *Botrytis cinerea* infections	[54]
*Capsicum annuum*	*CaAP2/ERF064*	AP2/ERF	Enhances resistance to Phytophthora blight in *Capsicums*	[55]
*Triticum aestivum*	*TaAP2-15*	AP2	Increases susceptibility to *Puccinia striiformis f.* Sp. *Tritici* in wheat	[56]
*Solanum tuberosum*	*StERF94*	ERF	Inhibits fungal proliferation in cellular tissues	[57]
*Solanum tuberosum*	*StERF3*	ERF	Negatively regulates resistance to *Phytophthora* infestans	[58]
*Solanum lycopersicum*	*SlERF-B3*	ERF	Differentially expressed in resistant and susceptible tomato during TYLCV infection	[59]
*Nicotiana tabacum*	*NtERF5*	ERF	Enhances resistance to tobacco mosaic virus	[60]
*Vitis vinifera*	*VvRAV1*	RAV	Suppresses the host’s defense responses and facilitates the accumulation and infection of *glrav-2*	[61]
*Glycine max*	*GmERF54*	ERF	Decreases resistance to CCW	[62]
*Solanum lycopersicum*	*Pti5*	ERF	Contributes to resistance to potato aphid and *Botrytis cinerea* in tomato	[63,64]
*Brassica rapa*	*BrERF11b*	ERF	Enhances plant resistance to chewing insects and sap-sucking insects	[65]
*Malus × domestica*	*MdERF11*	ERF	Enhances resistance to gray mold disease in apple trees	[66]
*Solanum lycopersicum*	*SlERF01*	ERF	Enhances resistance to *Stemphylium lycopersici* in tomato	[67]
*Vitis amurensis*	*VaERF16*	ERF	Improves resistance to *Botrytis cinerea* in grape	[68]
*Capsicum annuum*	*CaERF1A*	ERF	Enhances resistance to necrotrophic fungal pathogens	[69]
*Oryza sativa*	*OsERF83*	ERF	Improves resistance to rice blast disease	[70]
*Arabidopsis thaliana*	*ORA59*	ERF	Enhances resistance to necrotrophic pathogen infection	[71]
*Arabidopsis thaliana*	*AtERF6/72*	ERF	Enhance resistance to *Botrytis cinerea*	[72,73]
*Oryza sativa*	*OsEREBP1*		Enhances disease resistance	[74]
*Glycine max*	*GmERF113*	ERF	Enhances immune responses to *Phytophthora sojae*	[75]
*Populus tremula*	*ERF139*	ERF	Increases lignin synthesis and defensive capacity of cell wall	[76]
*Arabidopsis thaliana*	*AtERF114*	ERF	Increases lignin synthesis and defensive capacity of cell wall	[77]
*Malus × domestica*	*MdERF114*	ERF	Increases lignin synthesis and defensive capacity of cell wall	[48]
